# Single-Cell Selective Retrieval Method Using Cone-Shaped Light-Responsive Gas-Generating Polymer Microscaffold Array Chip

**DOI:** 10.3390/s26092687

**Published:** 2026-04-26

**Authors:** Hidetaka Ueno, Yoshinori Akagi, Shohei Yamamura

**Affiliations:** 1 Center for Advanced Medical Engineering Research & Development (CAMED), Kobe University, 1-5-1 Minatojima-minamimachi, Chuo-ku, Kobe 650-0047, Hyogo, Japan; 2 R&D Center Corporate Advanced Technology Institute Life Science Development Center, Sekisui Chemical Co., Ltd., 2-1 Hyakuyama, Shimamoto-cho 618-0021, Osaka, Japan; 3 Health and Medical Research Institute, National Institute of Advanced Industrial Science and Technology (AIST), 2217-14 Hayashi-cho, Takamatsu 761-0395, Kagawa, Japan

**Keywords:** light-responsive gas-generating polymer, single-cell retrieval, single-cell analysis, heterogeneity, cell chip

## Abstract

The detection and retrieval of specific single cells within a cell population is useful for elucidating cellular function as well as early-stage cancer diagnosis by detecting circulating tumor cells. Microcapillaries are used to retrieve specific single cells from cell populations; however, quick single-cell retrieval that firmly adheres to the substrate without damaging the cell is difficult. In this study, we propose a single-cell selective retrieval method using a cone-shaped light-responsive gas-generating polymer (LGP) microscaffold array chip. An LGP microscaffold array chip with cone-shaped LGP microscaffolds was fabricated without any special equipment. When human cervical cancer cells were spread on the LGP microscaffold array chip, adhesion was achieved, and single cells were arranged on up to 73.3% of the cone-shaped LGP microscaffolds. When low-toxicity ultraviolet A light was irradiated from the back of the LGP microscaffold array chip, only a single cell adhering to the cone-shaped LGP microscaffold was released by the generated N_2_ gas bubbles. More than 90% of the retrieved cells adhered, spread, and could be cultured for over 24 h. In conclusion, the proposed method is a simple and quick single-cell retrieval method that requires only a conventional inverted fluorescence microscope.

## 1. Introduction

Cell retrieval is a method for detecting, collecting, and moving specific cells from a cell population to different places for experiments, such as genetic analysis, and is necessary for various biotechnologies, including drug discovery and regenerative medicine [1,2,3,4,5,6]. In recent years, selective retrieval methods collecting a specific target single cell from a cell population have been developed to elucidate the heterogeneity of the cell population at the single-cell level [7,8,9]. Additionally, selective retrieval of the target single cell is essential for cloning cells that express specific functions, genetic analysis of rare cells, and development of more accurate diagnostic techniques based on these findings [10,11,12,13]. For example, research on the detection of circulating tumor cells (CTCs) has been conducted. Specifically, the number of CTCs in blood samples has been measured using microarrays and microfluidic devices. These methods can measure the number of CTCs; however, the detailed characteristics of CTCs, such as their malignancy and metastatic ability, remain difficult to evaluate. Therefore, a method of collecting CTCs for genetic analysis is required. Still, harmlessly collecting only the target single cell is challenging [14,15,16,17,18,19].

Micromanipulators have been developed for selective single-cell retrieval [20,21]. In such cases, the tip of a hollow microcapillary is placed near the target single cell, which is aspirated along with the surrounding culture medium. Long-term cultivation of the retrieved cells individually will enable the characterization of rare cells, including CTCs, using time-lapse data. However, collecting cells that firmly adhere to substrates, such as glass slides or microplates, is difficult. Furthermore, if high suction pressure is used to aspirate firmly adhered cells, a risk of damage to the cells exists owing to shear stress. Therefore, to collect firmly adhered cells, they must be released from the substrate before aspiration.

Methods for releasing cells from the substrate, such as proteolytic enzymes, thermoresponsive polymers, and photodegradable hydrogel polymers, have been used [22,23,24,25,26]. However, releasing a single target cell from a cell population and retrieving it selectively and quickly is challenging. Moreover, when using chemical materials such as thermoresponsive and photodegradable hydrogels and their chemical reactions, the surface condition of the substrate to which the cells adhere is partially limited. Thus, forming the most suitable cell-adhesion surface for each cell type using coating materials such as fibronectin is difficult [27,28,29]. Therefore, a simple and selective single-cell retrieval method that imposes less stress on the cell and does not depend on chemical reactions is required.

In our research group, we proposed a single-cell retrieval method using a light-responsive gas-generating polymer (LGP) microarray that generated N_2_ gas bubbles when irradiated with low-toxicity ultraviolet A (UVA) light [30]. Cells adhered to the LGP microarray were physically released by the N_2_ gas bubbles generated from the LGP; therefore, the LGP surface could be coated with the most suitable material, such as an extracellular matrix (ECM), for each cell. Furthermore, even cells firmly adhered to by the coating agent could be released at a 100% ratio within a few seconds of light irradiation. In addition, because LGP was integrated on the chip, connecting external equipment, such as electrical wires or microtubes, to the chip was not necessary. Moreover, in conventional cell manipulation techniques using bubbles, a powerful laser was used to generate the bubbles using heat. Yet, when using LGP, bubbles could be generated using a normal inverted microscope without heating [31,32,33]. However, the size of the LGP microarray was not optimized for single-cell size, and the percent of a single cell adhering to one LGP microarray was at most 12.47 ± 3.90%. Furthermore, an issue occurred with the low collection efficiency of single cells per unit area because of the low integration density of LGP microarrays.

In this study, we propose a highly efficient single-cell retrieval method using a cone-shaped LGP microscaffold with a size such that only a single cell can adhere. The proposed LGP microscaffold array chip was fabricated using a transfer process. First, the fabrication accuracy of cone-shaped LGP microscaffolds was evaluated. Subsequently, multiple human cervical cancer (HeLa) cells were adhered to the surface of a cone-shaped LGP microscaffold coated with fibronectin. Using fluorescence microscopy, the percent of single-cell adhesion was calculated by observing the surface of the LGP microscaffold array chips. Subsequently, by generating N_2_ gas bubbles from the cone-shaped LGP microscaffold where a single cell was adhered, we selectively released single cells and calculated the percent of single-cell release, which was the percent of single cells released among adherent cells. The released cells were then transferred to a common 96-well plate for cell culture. By evaluating the release efficiency and damage to cells, we demonstrated the usefulness of the single-cell retrieval method using the LGP microscaffold array chip.

## 2. Principle of Proposed Method

**Figure 1:**
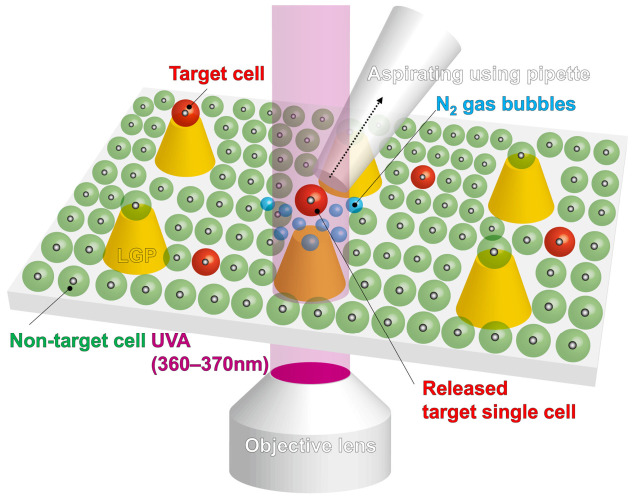
Principle of a single-cell retrieval method using an LGP microscaffold array chip. Cells spread on a cone-shaped LGP microscaffold were observed and evaluated using an inverted fluorescence microscope. After deciding on the target single cell, an objective lens of the inverted fluorescence microscope was moved under the target cell on the cone-shaped LGP microscaffold. Subsequently, the filter of the inverted fluorescence microscope was changed to irradiate UVA light. Between 1 and 10 s, N_2_ gas bubbles were generated from the cone-shaped LGP microscaffold after changing the excitation light to UVA light. The target cells were selectively released from the surface of the cone-shaped LGP microscaffold by the N_2_ gas bubbles and aspirated using a pipette.

Figure 1 shows a schematic of the single-cell retrieval method using an LGP microscaffold array chip, in which multiple cone-shaped LGP microscaffolds are arranged in an array. The cone-shaped LGP microscaffold, which has a top surface such that only a single cell could adhere to it, is arranged at equal intervals in an array on a transparent substrate. The surface of the cone-shaped LGP microscaffold, where the cells adhere, can be coated with the most suitable coating materials for each cell type, so that the cells to be seeded can adhere easily and are less damaged. In the proposed process, the cells are spread and adhered to the cone-shaped LGP microscaffold coated with the appropriate material. Then, the surface of the LGP microscaffold array chip where the cells adhered is observed using a normal inverted fluorescence microscope. The target cell to be retrieved is determined. By positioning the objective lens of the inverted fluorescence microscope under the target cell and switching the fluorescence filter to irradiate UVA (360–370 nm) light, N_2_ gas bubbles are generated from the cone-shaped LGP microscaffold where the target cell adhered. The adhesion of the cone-shaped LGP microscaffold to a single cell is inhibited by the N_2_ gas bubbles, and the cells are released. The released cells are collected using a normal pipette.

## 3. Materials and Methods

### 3.1. Cell Culture and Staining

HeLa-H2B-green fluorescent protein (GFP) cells were used as the culture cell lines. HeLa cells are human cervical cancer cells that express GFP in the nucleus. The HeLa cell culture was performed using methods shown in previous research [30]. To observe the shape of the cells, the membranes of HeLa cells were stained with Cell Bright Green (30021; Biotium, Inc., Fremont, CA, USA).

### 3.2. Fabrication Method for LGP Microscaffold Array Chip

**Figure 2:**
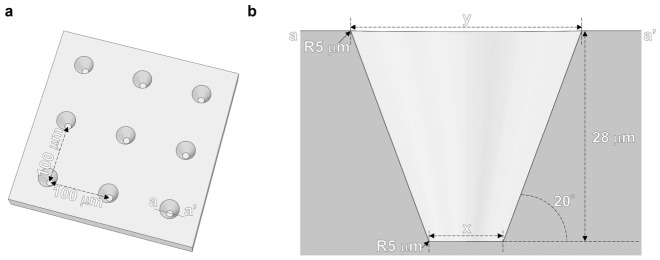
Design of the PS microchamber array chip. (**a**) Microchambers were arranged in a grid pattern. The distance between microchambers was 100 μm. (**b**) The cross-section corresponds to the a–a′ line indicated in (**a**), representing a sectional view of the microchamber. The microchamber was a tapered shape. Two sizes of microchambers were designed. The bottom diameter of microchamber: x was 11 μm for Design_1 and 16 μm for Design_2. The top diameter of microchamber: y was 31 μm for Design_1 and 36 μm for Design_2. The microchamber depth was 28 μm. The edges of the microchamber have a radius of curvature of 5 µm.

An LGP microscaffold array chip was fabricated using a polystyrene (PS) microchamber array chip made by injection molding. The design of the PS microchamber array chip used for the transfer is shown in Figure 2. On the PS microchamber array chip of 76 mm × 26 mm size, multiple microchambers were fabricated at 100 µm intervals (Figure 2a). The microchamber was conical with a 20° inclined structure to pull out the mold during injection molding. In this study, microchambers of two different sizes were fabricated. The diameters of the top of the microchamber were 31 and 36 µm. Furthermore, the diameters of the bottom of the microchamber were 11 and 16 µm. The depth of the microchamber was 28 µm for both designs (Figure 2b).

**Figure 3:**
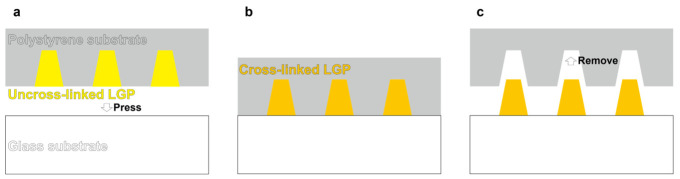
Fabrication process of the LGP microscaffold array chip. (**a**) Pressing the PS microchamber array chip embedded LGP on a glass slide substrate. (**b**) Carrying out the polymerization reaction of the LGP at room temperature of 22 °C for 24 h. (**c**) Removing the PS microchamber array chip and creating cone-shaped LGP microscaffolds on the glass slide.

The LGP microscaffold array chip was fabricated by transferring LGP from the PS microchamber array chip onto a glass substrate. The fabrication process of the LGP microscaffold array chip is illustrated in Figure 3. First, the main and crosslinking agents of LGP were mixed in a volume ratio of 20:1. Then, LGP was filled into the microchamber. Before crosslinking, the sample was pressed against a glass substrate (S2445; Matsunami Glass Ind., Ltd., Kishiwada, Japan). By leaving it for 24 h at room temperature of 22 °C, LGP was crosslinked. Finally, the cone-shaped microscaffold was transferred to a glass substrate by removing the PS microchamber array chip from the substrate.

The fabricated cone-shaped LGP microscaffold with a convex LGP structure was observed using a scanning electron microscope (SEM, JSM-6060-EDS, JEOL Ltd., Tokyo, Japan) and a white light interferometer (NT91001A-in motion, Bruker, Billerica, MA, USA). In addition, the arithmetic mean height (Ra) and maximum height of the profile (Rt), which represented the surface roughness of the LGP microscaffold, were calculated [34].

### 3.3. Fluorescence Observation

Fluorescence images were captured to evaluate the autofluorescence of the cone-shaped LGP microscaffold and glass surface after spreading the HeLa cell suspension onto the LGP microscaffold array chip. The surfaces of the cone-shaped LGP microscaffolds and HeLa cells were observed using a fluorescence microscope (IX-73, EVIDENT CO., Tokyo, Japan) and a charge-coupled device (CCD) camera (DP80; EVIDENT CO., Tokyo, Japan). Because HeLa cells expressing GFP were used, the intensity of HeLa cells was measured using a fluorescence image taken with a fluorescence filter (U-FBNA; ex: 470–495 nm, em: 510–550 nm; EVIDENT CO., Tokyo, Japan). The fluorescence intensity was measured by image analysis using ImageJ software (v1.53t, National Institutes of Health (NIH), Bethesda, MD, USA). For each condition, fluorescence intensities were acquired from three independent locations, and the mean and standard deviation were determined.

### 3.4. Selective Single-Cell Retrieval from LGP Microscaffold Array Chip

**Figure 4:**
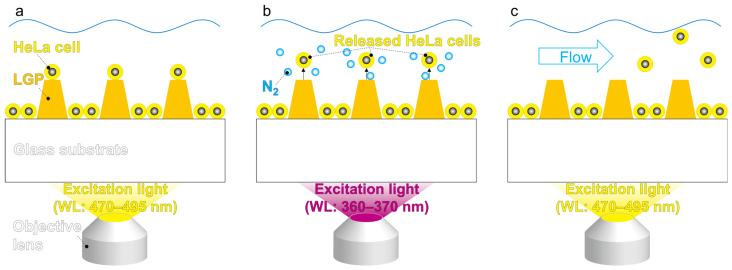
Single-cell retrieval process using the LGP microscaffold array chip. (**a**) Observing single cells adhered on the cone-shaped LGP microscaffolds and deciding the target cells using 470–495 nm excitation light. (**b**) Releasing adhered single cells on LGP microscaffolds by generated N_2_ gas bubbles using 360–370 nm excitation light. (**c**) Collecting released single cells using a normal pipette while observing the surface of the LGP microscaffold array chip using 470–495 nm excitation light.

Single HeLa cells that adhered to the LGP microscaffold array chip were collected and transferred to the chamber of a 96-well plate for observation, and the proposed single-cell retrieval method was evaluated. The single-cell retrieval process is illustrated in Figure 4. This protocol is similar to our former research [30]. First, silicone rubber sheets (length: 20 mm, width: 3 mm, thickness: 0.5 mm) were adhered at 5 cm intervals to both ends of the LGP microscaffold array chip using dimethylpolysiloxane (PDMS; SILPOT 184, Dow Corning Toray Co., Ltd., Tokyo, Japan). A cover glass (No. 2, Matsunami Glass Ind., Ltd., Kishiwada, Japan) was placed over the LGP microscaffold array chip, and a 1 mL solution of fibronectin at a concentration of 20 µg/mL was introduced into the gap between the chip and cover glass. It was left overnight in a refrigerator set at 4 °C. After washing the LGP microscaffold array chip with PBS, the LGP microscaffold array chip was covered with a new cover glass (No. 2, Matsunami Glass Ind., Ltd., Kishiwada, Japan), and a 1 mL suspension of HeLa cells was introduced into the gap between the chip and cover glass. The cell suspension was thoroughly pipetted to minimize the formation of cell aggregates. The cell suspension concentrations were 1 × 10^5^, 1 × 10^6^, and 5 × 10^6^/mL. After incubation for 1 h, the cover glass was removed, and HeLa cells that did not adhere to the LGP microscaffold array chip were washed with PBS. By this process, HeLa cells were adhered to the surface of a cone-shaped LGP microscaffold coated with fibronectin. Some of the attached cells were randomly selected as the target cells. The LGP microscaffold array chip was placed in a Petri dish (S90-NC18, Fine Plus International, Kyoto, Japan) containing 15 mL of medium. HeLa cells, adhered to the cone-shaped LGP microscaffolds, were observed using an inverted fluorescence microscope (IX-73, EVIDENT CO., Tokyo, Japan) and a CCD camera (DP80, EVIDENT CO., Tokyo, Japan) (Figure 4a). A fluorescence filter (U-FBNA) that emitted excitation light at 470–495 nm was used for cell observation. The percent of single cells adhering to the observed cone-shaped LGP microscaffolds was calculated as follows:

(1) $$\mathrm{Percent}\;\mathrm{of}\;\text{single-cell}\;\mathrm{adhesion}\;=\;\frac{\mathrm{Number}\;\mathrm{of}\;\mathrm{single}\;\mathrm{cells}\;\mathrm{adhered}\;\mathrm{to}\;\text{cone-shaped}\;\mathrm{LGP}\;\mathrm{microscaffold}}{\mathrm{Number}\;\mathrm{of}\;\text{cone-shaped}\;\mathrm{LGP}\;\mathrm{microscaffolds}}\;\times \;100$$

After observing the adhered HeLa cells using fluorescence microscopy, the fluorescence filter (U-FBNA) was changed to another filter (U-FUNA; ex: 360–370 nm, em: 420–460 nm). By irradiating UVA light with a wavelength of 360–370 nm, N_2_ gas bubbles were generated from the cone-shaped LGP microscaffold. The generated N_2_ gas bubbles released single cells that adhered to the cone-shaped LGP microscaffold (Figure 4b). The percent of single-cell release, which indicated the percent of single cells released among the cells that adhered to the cone-shaped LGP microscaffold, was calculated as follows:

(2) $$\mathrm{Percent}\;\mathrm{of}\;\text{single-cell}\;\mathrm{release}\;=\;\frac{\mathrm{Number}\;\mathrm{of}\;\mathrm{single}\;\mathrm{cells}\;\mathrm{released}\;\mathrm{from}\;\text{cone-shaped}\;\mathrm{LGP}\;\mathrm{microscaffold}\;}{\mathrm{Number}\;\mathrm{of}\;\mathrm{single}\;\mathrm{cells}\;\mathrm{adhered}\;\mathrm{to}\;\text{cone-shaped}\;\mathrm{LGP}\;\mathrm{microscaffolds}}\;\times \;100\;$$

The released single cells were collected using a commercially available pipette (Gilson, Middleton, WI, USA) and transferred to a centrifuge tube (339650; Thermo Fisher Scientific, Waltham, MA, USA) (Figure 4c). After centrifuging the medium containing the collected HeLa cells (700 rpm, 3 min) and removing the excess medium, 200 μL of the new medium was added to the centrifuge tube to prepare a cell suspension. The cell suspension was then transferred to a 96-well plate (165305; Thermo Fisher Scientific). The single cells collected in the 96-well plate were cultured in a CO_2_ incubator (37 °C, 5% CO_2_; MCO-18AIC, Sanyo Electric, Osaka, Japan). The collected HeLa cells were observed after 24 h using a fluorescence microscope (IX-73, EVIDENT CO., Tokyo, Japan) and a CCD camera (DP80, EVIDENT CO., Tokyo, Japan).

## 4. Results and Discussion

### 4.1. Fabrication of LGP Microscaffold Array Chip

**Figure 5:**
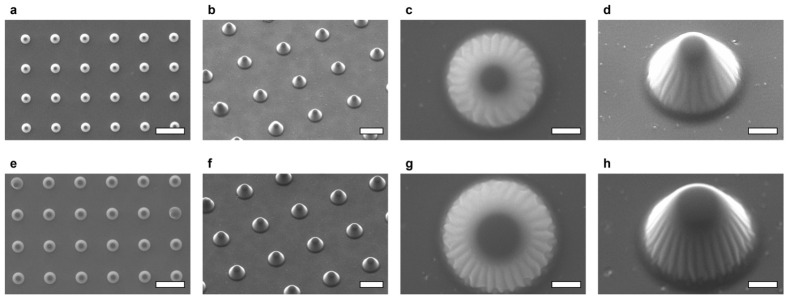
SEM images of the fabricated LGP microscaffold array chip. (**a**) Top view of arrayed cone-shaped LGP microscaffolds (Design_1). Scale bar indicates 100 μm. (**b**) Overview of arrayed cone-shaped LGP microscaffolds (Design_1). Scale bar indicates 50 μm. (**c**) Top view of arrayed cone-shaped LGP microscaffold (Design_1). Scale bar indicates 10 μm. (**d**) Magnification view of cone-shaped LGP microscaffold (Design_1). Scale bar indicates 10 μm. (**e**) Top view of arrayed cone-shaped LGP microscaffolds (Design_2). Scale bar indicates 100 μm. (**f**) Overview of arrayed cone-shaped LGP microscaffolds (Design_2). Scale bar indicates 50 μm. (**g**) Top view of arrayed cone-shaped LGP microscaffold (Design_2). Scale bar indicates 10 μm. (**h**) Magnification view of cone-shaped LGP microscaffold (Design_2). Scale bar indicates 10 μm.

The cone-shaped LGP microscaffolds were observed by SEM. The SEM images of the fabricated cone-shaped LGP microscaffolds are shown in Figure 5. Figure 5a–d show the cone-shaped LGP microscaffolds fabricated using the microchambers of Design_1. Figure 5e–h show the cone-shaped LGP microscaffolds fabricated using the microchambers in Design_2. The cone-shaped LGP microscaffolds of both designs were fabricated at equal intervals on glass substrates. Each cone-shaped LGP microscaffold had a tapered structure similar to that of the microchamber used for the fabrication, and a flat surface was observed at the top. Because the cone-shaped LGP microscaffold had a conical shape similar to that of the microchamber mold, the microchamber shape on the polystyrene substrate could be successfully transferred onto the glass substrate using the proposed fabrication method.

**Figure 6:**
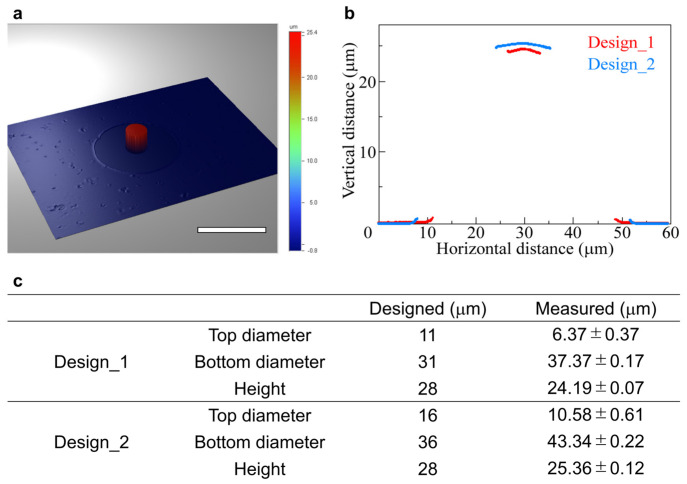
Shape of fabricated cone-shaped LGP microscaffold. (**a**) 3D profile of cone-shaped LGP microscaffold. Scale bar indicates 30 μm. (**b**) Measured cross-section of cone-shaped LGP microscaffold. (**c**) Design and measured size of cone-shaped LGP microscaffold (*n* = 3).

The cone-shaped LGP microscaffold was measured using a white light interferometer. A 3D profile of the measured shape is shown in Figure 6a. A flat surface was observed in the 3D profiles of both designs. However, measurement data could not be obtained for the tapered parts because the reflected light could not be obtained. The measurement results for the cross-sectional parts of the cone-shaped microscaffolds for the two designs are shown in Figure 6b. The horizontal axis represents the distance parallel to the surface of the LGP microscaffold array chip. The vertical axis represents the distance in the vertical direction from the surface of the LGP microscaffold array chips. In both Design_1 and Design_2, a planar surface on which the cells could adhere was observed on the cone-shaped LGP microscaffold. On the other hand, the tapered parts were not observed. The sizes of the cone-shaped LGP microscaffolds for each design, calculated from the measured sizes, are shown in Figure 6c. The sizes of the top and bottom diameters were calculated using the point where the measurement data were interrupted owing to the tapered structure. The top diameter was smaller, whereas the bottom diameter was larger than the designed size of the microchamber. This indicated that the fabrication error was caused by the curvature of the edge of the microchamber.

The height of the cone-shaped LGP microscaffold was approximately 3 µm smaller than the designed size. Additionally, from Figure 5c,d,g,h, wrinkles were observed in the tapered part. These wrinkles were considered to have occurred because LGP partially shrank when crosslinked. Therefore, the reason why the height of the cone-shaped LGP microscaffold was lower than the microchamber size was thought to be due to the shrinkage of LGP.

In Design_1, the sizes indicating surface roughness, Ra and Rt, were 0.11 and 0.50 µm, respectively, and in Design_2, Ra and Rt were 0.13 and 0.57 µm, respectively. The values of Ra and Rt were sufficiently small, less than 1 µm, and because the cell size was 10–30 µm, this indicated that it did not interfere with cell observation. Therefore, a cone-shaped LGP microscaffold with a flat surface suitable for single-cell adhesion and microscopy could be fabricated without the use of special equipment.

In this study, we fabricated LGP microscaffolds with two different sizes. Although the demonstration with only two types of LGP microscaffolds is insufficient to establish the applicability of the proposed method to arbitrary geometries, it nevertheless demonstrates that the proposed fabrication process could be used regardless of the size of the LGP microscaffold. This indicated that the optimal size of the LGP microscaffolds could be fabricated according to the cell type. Considering the size of HeLa cells, the diameter of which was less than 10 µm when not adhering, we considered that the size of Design_1 was appropriate for the adhesion of one HeLa cell, and Design_1 was used in the retrieval experiment of HeLa cells.

### 4.2. Fluorescence Imaging of HeLa Cells on LGP Microscaffold Array Chip

**Figure 7:**
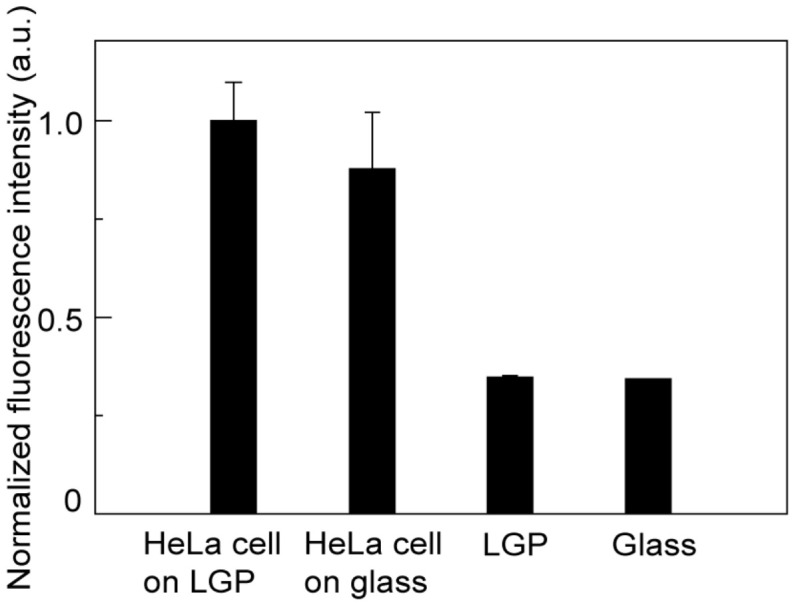
Fluorescence intensity of HeLa cells, cone-shaped LGP microscaffold, and glass slide.

To evaluate the compatibility of the LGP microscaffold array chip with fluorescence observations, cone-shaped LGP microscaffolds and GFP-expressing HeLa cells were irradiated with 470–495 nm excitation light using a fluorescence filter (U-FBNA) and observed using a fluorescence microscope. The HeLa cells that adhered to the cone-shaped LGP microscaffold and those that adhered to the glass substrate were clearly observed using an inverted fluorescence microscope. The normalized fluorescence intensities obtained from the fluorescence images are shown in Figure 7. More than a two-fold difference in fluorescence intensity existed between HeLa cells or not. In addition, the autofluorescence of the cone-shaped LGP microscaffold and glass surface was approximately the same. Autofluorescence becomes noisy during the fluorescence observation of cells and inhibits automatic observation of cells [35,36]. Therefore, the substrate and structure to which cells adhere should have sufficiently low autofluorescence. Because the autofluorescence of the cone-shaped LGP microscaffold fabricated in this study was sufficiently small compared to the fluorescence emitted by HeLa cells to be ignored, the autofluorescence of LGP did not affect the fluorescence observation of cells. Therefore, LGP is a suitable material for fluorescence measurements.

### 4.3. Single-Cell Selective Retrieval Using LGP Microscaffold Array Chip

**Figure 8:**
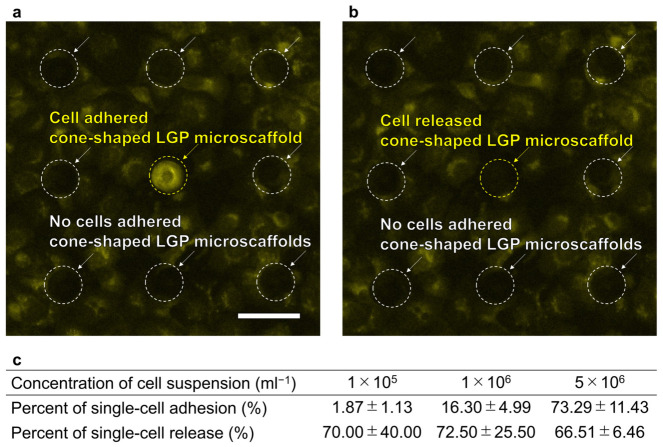
Fluorescence images of single HeLa cells adhered to and released from the cone-shaped LGP microscaffolds. (**a**) Single HeLa cells adhered to the LGP microscaffold. Scale bar indicates 100 μm. (**b**) After irradiating with UVA light, single HeLa cell was released from the LGP microscaffold. (**c**) Percent of single-cell adhesion and release at each concentration of cell suspension.

HeLa cells were spread on a cone-shaped LGP microscaffold surface coated with fibronectin and observed using a fluorescence filter (U-FBNA). A fluorescence image of the LGP microscaffold array chip after HeLa cell spreading is shown in Figure 8a. A single HeLa cell adhered to the cone-shaped LGP microscaffold at the center of the image, and its fluorescence intensity was more than twice that of the cone-shaped LGP microscaffold without adhered HeLa cells. After observation, the fluorescence filter was changed to U-FUNA (ex: 360–370 nm, em: 420–460 nm), and an excitation light of 360–370 nm was irradiated to generate N_2_ gas bubbles. The HeLa cells were released using N_2_ gas bubbles and collected using a pipette (Gilson, USA). A fluorescence image of the cone-shaped LGP microscaffold array after N_2_ gas bubble generation is shown in Figure 8b. Only the HeLa cells on the LGP microscaffold were released and collected.

Three different concentrations of the cell suspension were used for HeLa cell spreading. After the HeLa cells adhered, 266–282 cone-shaped LGP microscaffolds were used to calculate the percent of single-cell adhesion and release. There were no cone-shaped LGP microscaffolds attached by multiple HeLa cells. This may be attributed to the sufficiently small size of the cone-shaped LGP microscaffold, which likely prevents the attachment of multiple cells. The percent of single-cell adhesion and single-cell release were calculated using Equations (1) and (2), respectively, and are shown in Figure 8c. The percent of single-cell adhesion correlated with the concentration of the cell suspension, ranging from approximately 1.9% to 73.3%. When a cell suspension concentration of more than 5 × 10^6^/mL was used, the cells were stacked in multiple layers, making observation difficult, and accurate evaluation of cell occupancy was not possible. Therefore, 5 × 10^6^/mL was considered the optimal condition for achieving a percent of single-cell adhesion of over 70%.

The percent of single-cell release was approximately 66.5–72.5%. No significant difference existed in the concentration of each cell suspension, indicating that the adhered single cells could be reliably released at a constant ratio. However, unlike in previous studies, the percent of single-cell release was not 100% [30]. This was thought to be because the cone-shaped LGP microscaffold fabricated in this study was smaller than those used in previous studies, and the generated N_2_ gas bubbles passed through without reliably contacting the adhered cells, or because the wrinkles on the surface increased the adhesion strength of the cells.

The cone-shaped LGP microscaffold array chip proposed in this study enabled a higher integration density of the cone-shaped LGP microscaffold compared to previous studies [30]. Concretely, at least 180,000 cone-shaped LGP microscaffolds were fabricated on a single chip (Maximum usable area, considering handling constraints: 75 mm × 25 mm). When conducting the retrieval process with a cell suspension of 5 × 10^6^ cells/mL, the cone-shaped LGP microscaffold array chip would achieve single-cell retrieval of approximately 90,000 cells. This represents a recovery efficiency more than 15 times higher than that of the microarray chip used in our previous study (Table 1). Although the required number of cells for analysis differs for each experiment, the cone-shaped LGP microscaffold array chip provided sufficient capacity for general single-cell analysis.

In this study, cone-shaped LGP microscaffolds were coated with fibronectin [37]. Despite the fibronectin coating on the cone-shaped LGP microscaffold surfaces, HeLa cells were rapidly released by the N_2_ gas bubbles generated from the LGP microscaffolds. These results demonstrate that the cell release process remains unaffected by coating materials such as the ECM, which is consistent with findings from previous studies [38]. In our previous study, the surface of LGP was coated with fibronectin, collagen, and poly-D-lysine. Cells on these coated surfaces not only adhered well but were also efficiently released from the LGP surface.

**Figure 9:**
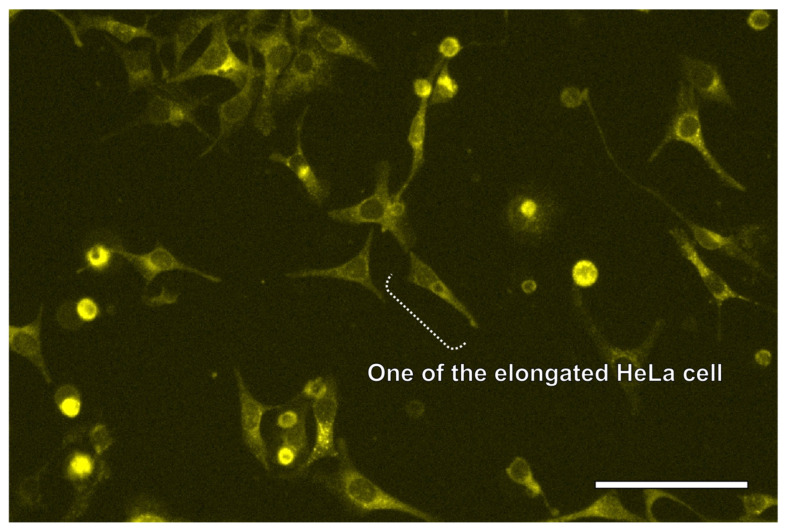
Fluorescence image of HeLa cells retrieved from the cone-shaped LGP microscaffold array chip. Scale bar indicates 20 μm.

The retrieved HeLa cells were then transferred to a 96-well plate and cultured following cell retrieval. Figure 9 shows the fluorescence images of the retrieved cells after 24 h. Multiple adhered and spread HeLa cells were observed at the bottom of the 96-well plate chambers. Given that more than 90% of the cells adhered to and elongated, the retrieved cells did not suffer significant damage during the proposed cell retrieval process. In the proposed method, UVA exposure and N_2_ bubbles could potentially damage cells. Previous studies have shown that this wavelength, commonly used for fluorescence observation, did not cause significant cellular damage [30,38]. Furthermore, because N_2_ gas is chemically stable, it is unlikely to initiate chemical effects on cells. Regarding the physical effects of bubbles, shear stress at the bubble surface and shock waves from bubble bursting can exert loads on cells [39,40,41]. However, as the N_2_ bubbles only contacted the cells immediately after emergence from the cone-shaped LGP microscaffold, minimal shear stress was applied to the cells. In addition, in the proposed method, the bubbles move to the liquid surface at a sufficient distance from the cells immediately after generation and do not burst near the cells or generate shockwaves. Consequently, the proposed method is considered almost harmless, which means that no visible damage was observed to retrieved cells using a standard fluorescent microscopy, for single-cell retrieval. Although a quantitative evaluation of cell viability was not included in this study, qualitative observations indicated that cells remained viable throughout the experiments. A quantitative assessment will be conducted in future work to further validate these findings.

Applications of the proposed technology include the screening and analysis of rare single cells such as CTCs. We have previously developed a microarray chip for CTC detection and analysis [42]. We also developed a method to retrieve single cells using a micromanipulator for subsequent functional analysis [43,44]. In future work, to trap and fix single cells with a higher ratio and reliably release the trapped cell using N_2_ gas bubbles, the fabrication of an LGP structure with a concave shape instead of a convex shape will also be considered. Further, increasing the density of cone-shaped LGP microscaffolds, and integrating the cone-shaped LGP microscaffold array chip with existing micromanipulators [44] are expected to enable the retrieval of rare cells by automated operation with a higher recovery rate and less cellular damage in future work.

## 5. Conclusions

In this study, we proposed an efficient single-cell retrieval method using an LGP microscaffold array chip that generates N_2_ gas. Using cone-shaped LGP microscaffolds for single-cell attachment, HeLa cells were isolated individually, and selective, rapid release, and recovery of cells attached to the LGP microscaffolds were achieved using excitation light from a general inverted fluorescence microscope. Single cells were attached to over 70% of the LGP microscaffolds, and over 65% of these single cells were successfully released. The single-cell retrieval efficiency per chip was more than 15 times higher than that reported in our previous study. Furthermore, over 90% of the retrieved HeLa cells were elongated in the 96-well plate chambers, indicating that excitation light and N_2_ gas bubbles caused negligible cellular damage. The size and surface of the cone-shaped LGP microscaffold could be easily optimized for diverse cell types. Therefore, the proposed single-cell retrieval method using the LGP microscaffold array chip is technically simple, minimizes cell damage, and enables the highly selective isolation, analysis, and recovery of single cells using only a general inverted fluorescence microscope.

## Figures and Tables

**Table 1:** Comparison of the efficiency of selective single-cell retrieval.

	Previous Research [30]	This Research
Measured LGP scaffold top diameter [μm]	65.7	6.4
Measured LGP scaffold bottom diameter [μm]	110.2	37.4
Measured LGP scaffold height [μm]	48.0	24.2
Designed LGP scaffold interval [μm]	212	100
Density of LGP scaffold [/mm^2^]	25	100
Number of LGP scaffold capable of being created on one chip (75 × 25 mm)	46,875	187,500
Percent of single-cell adhesion [%]	12.5	73.3
Percent of single-cell release [%]	100	66.5
Number of single cells collected from one chip (75 × 25 mm)	5859	91,396

## Data Availability

Data are contained within the article.
